# Associations between Neonatal Serum Bilirubin and Childhood Obesity in Term Infants

**DOI:** 10.1038/s41598-019-51043-w

**Published:** 2019-10-10

**Authors:** Lile Zou, Huan Yu, Yuan He, Lijuan Luo, Wenbin Dong, Jun Zhang, Xiaoping Lei, Christian Wieg

**Affiliations:** 1 Department of Histology and Embryology, Southwest Medical University, Luzhou, Sichuan China; 2 Department of Neonatology, Affiliated Hospital of Southwest Medical University, Luzhou, Sichuan China; 3 MOE-Shanghai Key Laboratory of Children’s Environmental Health, Xinhua Hospital, Shanghai Jiao Tong University School of Medicine, Shanghai, China; 4 Birth Defects Clinical Medical Research Center of Sichuan Province, Luzhou, Sichuan China; 5 Department of Perinatology, Southwest Medical University, Luzhou, Sichuan China; 6 Department of Neonatology, Children’s Hospital Aschaffenburg, Aschaffenburg, Germany

**Keywords:** Neonatology, Paediatric research

## Abstract

Inverse correlations between serum bilirubin level and obesity had been reported in adults. We aimed to investigate the associations between neonatal hyperbilirubinemia and childhood obesity. Data was obtained from the U.S. Collaborative Perinatal Project (CPP), a multicenter study from 1959 to 1976. Data of serum bilirubin in term newborns were used to observe the association with obesity at age of 7 years. Logistic regression models were performed to calculate adjusted odds ratios (aORs) for obesity. For children from the same mother sharing similar factors, Generalized Estimating Equation (GEE) model was used to correct for intracluster correlation. Relative to newborns with total serum bilirubin (TSB) < 3 mg/dl, there are lower risks for obesity in those with 3 mg/dl ≤ TSB < 6 mg/dl (aOR 0.91; 95%CI 0.81, 1.02), 6 mg/dl ≤ TSB < 9 mg/dl (aOR 0.88; 95%CI 0.78, 0.99), 9 mg/dl ≤ TSB<13 mg/dl (aOR 0.83; 95%CI 0.71, 0.98). By stratifying for subtypes of bilirubin, the inverse correlations only existed in exposure to unconjugated bilirubin. By using the GEE model correcting for intracluster correlations, the results are consistent. In summary, exposure to bilirubin up to 13 mg/dl is inversely associated with obesity at the age of 7 years in term infants.

## Introduction

Obesity has reached epidemic proportions globally, and it has shown a marked increase over the past 4 decades. While global obesity rates were around 3% in adult men and just over 6% in adult women in 1975, 11% of men and 15% of women were obese in 2014. Thus, more than 600 million adults meet the criteria of obesity as a major contributor to the global burden of chronic disease and disability^1^. At the same time, the rate of childhood obesity is also rising and presenting one of the most serious global public health challenges. In 2014, an estimated amount of 41 million children under 5 years old were overweight or obese^2^. International Obesity Task Force has shown that one in 10 children, a total of 155 million globally, is overweight, and approximately 30–45 million of them are classified as obese, accounting for 2 to 3 percent of the world’s children population aged 5–17 years^3,4^. Globally, obesity is stronger linked to deaths than underweight^3^. According to World Health Organization, obesity is related up to 2 to 7 percent of the total health care costs in some developed countries^5^.

Bilirubin has toxic effects on developing neuronal tissues^6,7^, and is also a potent endogenous antioxidant and cytoprotectant^8–10^. The chronic, low-grade inflammation is widely reported to be involved in the mechanism of obesity^11,12^. An inverse relationship between serum bilirubin and the risk of cardio-metabolic disease has been reported previously in some cross-sectional studies^13–19^. So the hypothesis was generated that bilirubin has an protective effect relating to metabolic syndrome^13–17^, lipid metabolism disorder^18^, and hypertension^19^ due to its antioxidant properties. According to the developmental origins of health and disease (DoHaD) hypothesis, exposure to risk factors early-in-life can affect health status in later life^20^. However, almost all of the current studies on this topic are regarding adult populations exposed to low levels of serum bilirubin, as data from high level of bilirubin exposure in early life are lacking. Physiological jaundice is very common in newborns presenting much higher serum bilirubin concentrations than any other normal population. However, inconsistent with the previous studies on this topic^13–19^, our previous study demonstrated that neonatal bilirubin concentrations positively associated with childhood obesity among preterm infants^21^. So the question rise whether term-newborns with hyperbilirubinemia has the same associations with obesity in later life? Basing the U.S. Collaborative Perinatal Project (CPP) data, the purpose of this study is to exposure the potential associations between serum bilirubin in neonatal period and childhood obesity among term-newborns.

## Methods

### Study population

The data using in the current study obtained from the US. CPP, a multicenter birth cohort study from 1959 to 1966 contained 56 990 pregnancies within 46 021 women at 12 centers. By using standardized procedures, the trained observer measured and recorded anthropometric parameters of offspring (including weight, height, and etc) at each follow-up visit during 7 years follow-up time. At age of 7 years, the full-scale score (IQ) of Wechsler Intelligence Scale for Children (WISC) was administered^22^. The standard questionnaire in the cohort study recorded general characteristics of mothers and infants, including socioeconomic status, infant’s races, gestational ages, delivery methods and maternal ages, smoking, educational levels, prepregnancy body mass index (BMI), marital status, etc. The data have been publicly available at the U.S. National Archives (www.archives.gov/), with detailed description provided in other study^23^. The study protocol was approval by the Institutional Review Board of the Affiliated Hospital, Southwest Medical University. Due to the publicly available de-identified data, this study without informed consent.

**Figure 1:**
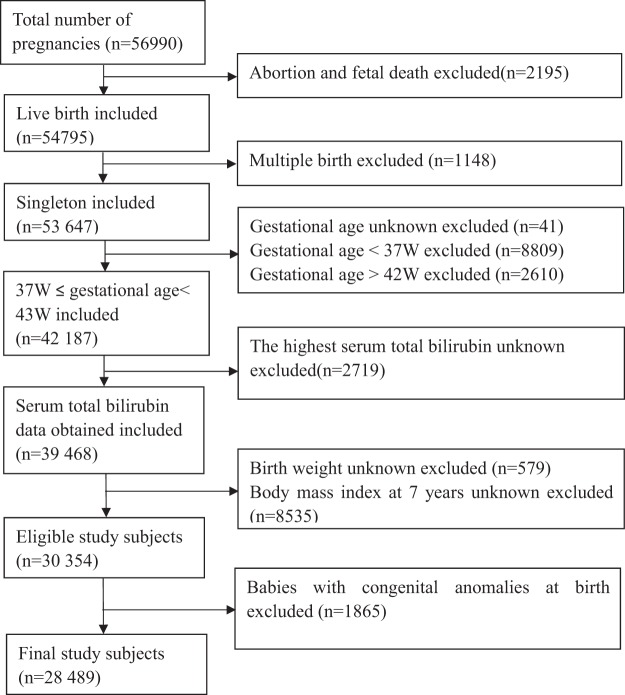
(Lei). Flow Chart in the Selection of Study Population from the U.S. Collaborative Perinatal Project Birth Cohort.

In this analysis, after stillbirths or terminations (*n* = 2195) and multiple births (*n* = 1148) were excluded, a total of 53 647 single births were enrolled. Next, we excluded newborns with gestational age at birth <37 weeks (*n* = 8809), ≥43 weeks (*n* = 2610) or unknown (*n* = 41). Infants with unknown serum bilirubin levels during neonatal period (*n* = 2719) or BMI at age 7 (*n* = 8535) have been also excluded. After excluding the infants with chromosomal congenital and severe structural anomalies, the final study subjects were left to be 28 489 term infants (Fig. 1).

### Bilirubin measurements

Except for direct spectrophotometry used in one center, the diazo method were used to measure bilirubin in the other 11 centers. Among the CPP laboratories, the coefficient of variation of standard specimens was about 10%; the intralaboratory coefficient of variation was about 2%^24^. This level of reliability is the same as the results in two more recent surveys of bilirubin measurements.

In the study protocol of the CPP, total serum bilirubin (TSB) levels were measured between 36 and 60 hours for each infant, as close to 48 hours as possible. If the value of TSB was exceeded 10 mg/dl (171μmol/L), the next test was performed 24 hours later. If the second TSB also higher than 10 mg/dl, a third measurement was obtained at 4 to 5 days of age. Additional bilirubin measurements were obtained at the decisions of physicians at each study site. We primarily used the maximum TSB as exposure factor and divided the babies into five groups based on the bilirubin concentrations: TSB < 3 mg/dl, 3 mg/dl ≤ TSB < 6 mg/dl, 6 mg/dl ≤ TSB < 9 mg/dl, 9 mg/dl ≤ TSB < 13 mg/dl, and TSB ≥ 13 mg/dl (as pathological jaundice^25^). In addition, the values of conjugated bilirubin (CB) could be obtained from 17150 infants in the CPP. Thus, we also used CB and unconjugated bilirubin (UCB) as independent factors to explore the correlations between the different type of bilirubin and pediatric obesity.

### Outcomes and confounders

Based on the childhood growth standards of WHO, obesity was referred as BMI for age and sex greater than 2 standard deviations above the WHO Growth ref.^26^. The validity and the reliability of the Wechsler Intelligence Scales for children have been confirmed elsewhere^27^. In this study, low IQ was referred to IQ < 70 at 7 years, and was considered as impaired neurodevelopment.

As described in our previous study^21^, perinatal factors were chosen as potential confounders: maternal characteristics included age at delivery (<20, 25–35, and >35 years), marital status (married, unmarried, and other), highest maternal education (<10th, 10th −12th, and >12th grades), maternal smoking (0, 1–19 and 20 cigarettes per day during pregnancy), hypertensive disorders during pregnancy (none, moderate, and severe), socioeconomic status (comprised of 5 categories as assessed by the original CPP investigators) and maternal pregnancy BMI (<18, 18–25, >25), BMI gain during pregnancy (<3, 3–6, >6). Infant characteristics included race (white, black, and other race); sex (male and female); gestational age (as categorical variable), delivery method (vaginal, cesarean section and others), birthweight (<2500 g, 2500g-4000g, and >4000 g); and feeding method (exclusively breast, exclusively bottle, mixed feeding, and unknown).

### Statistics analysis

The differences of the maternal and infantile demographic characteristics among different groups were compared by Cochran-Mantel-Haenszel Chi-square. Relative to the babies with neonatal TSB < 3 mg/dl, the crude odds ratios (ORs) for obesity and low IQ at 7 years old in each other group were calculated by the univariate logistic regression model (model 1). The adjusted ORs were calculated by a multivariable logistic regression model (model 2) to adjust the potential confounders. Because 3515 women, with 7619 pregnancies, contributed more than one birth, and the offspring of the same mother sharing the similar genetic and household factors, these intracluster correlations was corrected by using the Generalized Estimating Equation (GEE) model (model 3). All the statistical analyses were performed in SAS version 9.2 (SAS Institute, Cary, North Carolina).

### Ethical approval and informed consent

The study protocol was approval by the Institutional Review Board of the Affiliated Hospital, Southwest Medical University. Use of publicly available de-identified data is exempt from informed consent.

## Results

Table 1 showed the maternal and infantile baseline characteristics in different groups. Significant differences were observed in birthweight, gestational age, feeding methods, maternal smoking among term babies with different levels of TSB concentrations.

**Table 1:** Baseline Characteristics of Term Babies with Different Concentration of Total Serum Bilirubin.

Total Serum Bilirubin	<3 mg/dl (N = 5758)	≥3 mg/dl, <6 mg/dl (N = 9406)	≥6 mg/dl, <9 mg/dl (N = 8364)	≥9 mg/dl, <12 mg/dl (N = 3450)	≥13 mg/dl (N = 1511)	P
**Brithweight; N (%)**						<0.0001
<2500 g	249 (4.3)	390 (4.2)	443 (5.3)	242 (7.0)	167 (11.1)	
2500–4000 g	5072 (88.1)	8440 (89.7)	7562 (90.4)	3027 (87.7)	1264 (83.7)	
>4000 g	437 (7.6)	576 (6.1)	359 (4.3)	181 (5.3)	80 (5.3)	
**Male; N (%)**	2581 (44.8)	4510 (48.0)	4459 (53.3)	1877 (54.4)	898 (59.4)	<0.05
**Race; N (%)**						
White	3043 (52.9)	4838 (51.4)	4100 (49.0)	1698 (49.2)	837 (55.4)	<0.05
Black	2542 (44.2)	4271 (45.4)	3956 (47.3)	1605 (46.5)	614 (40.6)	
Others	173 (3.0)	297 (3.2)	308 (3.7)	147 (4.3)	60 (4.0)	
**Gestational age; N (%)**						<0.001
37W	302 (5.2)	648 (6.9)	781 (9.3)	407 (11.8)	279 (18.5)	
38W	648 (11.3)	1371 (14.6)	1528 (18.3)	636 (18.4)	302 (20.0)	
39W	1229 (21.3)	2161 (23.0)	2102 (25.1)	832 (24.1)	343 (22.7)	
40W	1558 (27.1)	2404 (25.6)	1957 (23.4)	793 (23.0)	293 (19.4)	
41W	1179 (20.5)	1646 (17.5)	1168 (14.0)	437 (12.7)	175 (11.6)	
42W	577 (10.0)	796 (8.5)	553 (6.6)	240 (7.0)	79 (5.2)	
43W	265 (4.6)	380 (4.0)	275 (3.3)	105 (3.0)	40 (2.7)	
**Feeding methods; N (%)**						<0.001
Exclusively breast	434 (7.5)	732 (7.8)	571 (6.8)	240 (7.0)	96 (6.4)	
Excessive bottle	4103 (71.3)	6948 (73.9)	6239 (74.6)	2398 (69.5)	999 (66.1)	
Mixed feeding	529 (9.2)	835 (8.9)	881 (10.5)	405 (11.7)	234 (15.5)	
Unknown	692 (12.0)	891 (9.5)	673 (8.1)	407 (11.8)	182 (12.1)	
**Maternal age; N (%)**						<0.001
<20	1140 (19.8)	2033 (21.6)	2003 (24.0)	830 (24.1)	324 (21.4)	
20–24	1949 (33.9)	3429 (36.5)	3044 (36.4)	1276 (37.0)	531 (35.1)	
25–29	1283 (22.3)	2065 (22.0)	1790 (21.4)	732 (21.2)	311 (20.6)	
30–34	828 (14.4)	1111 (11.8)	937 (11.2)	363 (10.5)	193 (12.8)	
≥35	558 (9.7)	768 (8.2)	590 (7.1)	249 (7.2)	152 (10.1)	
**Maternal smoking; N (%)**						<0.001
0	2690 (46.7)	4866 (51.7)	4738 (56.7)	2032 (58.9)	877 (58.0)	
1–19	1786 (31.0)	2884 (30.7)	2346 (28.1)	945 (27.4)	386 (25.6)	
≥20	1198 (20.8)	1525 (16.2)	1144 (13.7)	424 (12.3)	224 (14.8)	
Unknown	84 (1.5)	131 (1.4)	136 (1.6)	49 (1.4)	24 (1.6)	
**Socioeconomic status; N (%)**						0.32
1	380 (6.6)	637 (6.8)	653 (7.8)	237 (6.9)	96 (6.4)	
2	1535 (26.7)	2658 (28.3)	2432 (29.1)	1055 (30.6)	416 (27.5)	
3	1792 (31.1)	2896 (30.8)	2481 (29.7)	1024 (29.7)	474 (31.4)	
4	1270 (22.1)	1996 (21.2)	1684 (20.1)	669 (19.4)	320 (21.2)	
5	643 (11.2)	1035 (11.0)	949 (11.4)	376 (10.9)	171 (11.3)	
Unknown	138 (2.4)	184 (2.0)	165 (2.0)	89 (2.6)	34 (2.3)	
**Maternal BMI before pregnancy;**						0.08
<18.5	948 (16.5)	1497 (15.9)	1336 (16.0)	561 (16.3)	241 (16.0)	
18.5–25	3493 (60.7)	6007 (63.9)	5401 (64.6)	2183 (63.3)	950 (62.9)	
>25	1317 (22.9)	1902 (20.2)	1627 (19.5)	706 (20.5)	320 (21.2)	
**BMI gain during pregnancy; N (%)**						0.08
<3	1537 (26.7)	2660 (28.3)	2358 (28.2)	948 (27.5)	433 (28.7)	
3–6	3231 (56.1)	5304 (56.4)	4794 (57.3)	1980 (57.4)	827 (54.7)	
>6	975 (16.9)	1408 (15.0)	1183 (14.1)	509 (14.8)	246 (16.3)	
Unknown	15 (0.3)	34 (0.4)	29 (0.4)	13 (0.4)	5 (0.3)	

Abbreviations: BMI - body mass index.

There were 2406 obese children among out of 28 489 babies, with an overall incidence of 8.45%. Table 2 shows that, in term babies, relative to the group with neonatal TSB < 3 mg/dl, there are lower risks for obesity at age 7 years in babies with 3 mg/dl ≤ TSB<6 mg/dl (adjusted OR 0.91; 95% CI 0.81, 1.02), 6 mg/dl ≤ TSB < 9 mg/dl (adjusted OR 0.88; 95% CI 0.78, 0.99), 9 mg/dl ≤ TSB < 13 mg/dl (adjusted OR 0.83; 95% CI 0.71, 0.98), respectively. The incidence of obesity at age 7 years is not different comparing babies with TSB < 3 mg/dl and TSB ≥ 13 mg/dl (adjusted OR 1.04; 95% CI 0.85, 1.27). By using GEE model to correct intracluster correlations, the results remain unchanged.

**Table 2:** The Odds Ratios of Obesity at age 7 in Term Newborns with Different Concentrations of Total Serum Bilirubin.

Total serum bilirubin	n (%)	Obesity at 7 years old					
		Model 1^a^		Model 2^b^		Model 3^c^	
		OR	95%CI	OR	95%CI	OR	95%CI
<3 mg/dl	552 (9.6)	1		1		1	
≥3 mg/dl, <6 mg/dl	791 (8.4)	0.87	0.77, 0.97	0.91	0.81, 1.02	0.90	0.78, 1.01
≥6 mg/dl, <9 mg/dl	656 (7.8)	0.80	0.71, 0.90	0.88	0.78, 0.99	0.86	0.74, 0.98
≥9 mg/dl, <13 mg/dl	264 (7.7)	0.78	0.67, 0.91	0.83	0.71, 0.98	0.82	0.66, 0.97
≥13 mg/dl	143 (9.5)	0.99	0.81, 1.20	1.04	0.85, 1.27	1.03	0.83, 1.22

Abbreviations: OR, odds ratio; CI, confidence interval.

^a^Model 1: Crude odds ratios;

^b^Model 2: Sex, race, socioeconomic status, highest maternal education, maternal age, gestational age, marital status, maternal smoking, hypertensive disorders during pregnancy, birthweight, feeding methods, maternal pregnancy BMI, BMI gain during pregnancy and parity were adjusted;

^c^Model 3: Generalized Estimating Equation model basing the same factors as model 2.

Table 3 demonstrates that, in consistence with TSB, similar associations could be found after differing unconjugated (UCB) and conjugated serum bilirubin levels (CB) relating to childhood obesity at age 7 years only for UCB. Relative to the group with UCB < 3mg/dl, the adjusted ORs of obesity at age 7 years are 0.93 (95% CI 0.80, 1.09), 0.86 (95% CI 0.75, 1.00), 0.85 (95% CI 0.73, 0.99), and 1.12 (95% CI 0.89, 1.41) among babies with 3 mg/dl ≤ UCB < 6 mg/dl, 6 mg/dl ≤ UCB < 9 mg/dl, 9 mg/dl ≤ UCB < 12 mg/dl and UCB ≥ 13 mg/dl, respectively. On the contrary, comparing with babies with serum CB < 1 mg/dl, the adjusted ORs of obesity at age 7 years are 1.04 (95% CI 0.86, 1.26) and 1.26 (95% CI 1.01, 1.58) among babies with 1 mg/dl ≤ CB < 2 mg/dl and CB ≥ 2 mg/dl, respectively.

**Table 3:** The Odds Ratios of Obesity at age 7 in Term Newborns with Different Concentrations and Subtypes of Serum Bilirubin.

Bilirubin		Obesity at 7 years old						
		n (%)	Model 1		Model 2		Model 3	
			OR^a^	95%CI	OR^b^	95%CI	OR^c^	95%CI
Unconjugated bilirubin*	<3 mg/dl	324 (9.4)	1		1			
	≥3 mg/dl, <6 mg/dl	407 (8.6)	0.91	0.78, 1.06	0.93	0.80, 1.09	0.93	0.77, 1.09
	≥6 mg/dl, <9 mg/dl	314 (7.8)	0.81	0.69, 0.96	0.86	0.75, 1.00	0.85	0.72, 0.99
	≥9 mg/dl, <13 mg/dl	124 (7.7)	0.80	0.65, 0.99	0.85	0.73, 0 .99	0.84	0.73, 1.00
	≥13 mg/dl	115 (9.9)	1.05	0.84, 1.32	1.12	0.89, 1.41	1.11	0.88, 1.34
Conjugated bilirubin^#^	<1 mg/dl	1032 (8.2)	1		1		1	
	≥1 mg/dl, <2 mg/dl	148 (9.7)	1.19	0.99, 1.43	1.04	0.86, 1.26	1.04	0.85, 1.23
	≥2 mg/dl	104 (11.2)	1.40	1.13, 1.74	1.26	1.01, 1.58	1.23	1.01, 1.46

Model 1: Crude odds ratios;

Model 2: Race, sex, socioeconomic status, highest maternal education, maternal age, gestational age, marital status, maternal smoking, hypertensive disorders during pregnancy, birthweight, feeding methods, maternal pregnancy BMI, BMI gain during pregnancy and parity were adjusted;

Model 3: Generalized Estimating Equation model basing the same factors as model 2.

*Adjusted by conjugated bilirubin; ^#^Adjusted by unconjugated bilirubin.

In order to consider the influence of severe cholestatic and hemolytic pathology, in second step, we excluded infants with positive direct Coombs’ test, need exchange transfusion, CB ≥ 2 mg/dl, and definite hepatic disease at birth. Hence, the level serum TSB and UCB negatively correlated with body mass index at age 7 years (β = −0.18 and −0.24, respectively, *P* all <0.0001). To define possible negative influences of TSB concentrations on cognitive development we correlated TSB to IQ at age 7 years not finding any association (Table 4).

**Table 4:** The Odds Ratios of Low IQ (<70) at age 7 in Term Newborns with Different Concentrates of Serum Bilirubin.

Total serum bilirubin	n (%)	Obesity at 7 years old					
		Model 1^a^		Model 2^b^		Model 3^c^	
		OR	95%CI	OR	95%CI	OR	95%CI
<3 mg/dl	552 (9.6)	1		1		1	
≥3 mg/dl, <6 mg/dl	791 (8.4)	1.10	0.88, 1.36	1.00	0.80, 1.26	1.07	0.85, 1.29
≥6 mg/dl, <9 mg/dl	656 (7.8)	1.09	0.87, 1.36	0.95	0.75, 1.20	1.03	0.80, 1.26
≥9 mg/dl, <13 mg/dl	264 (7.7)	1.25	0.96, 1.63	1.06	0.81, 1.40	1.14	0.86, 1.41
≥13 mg/dl	143 (9.5)	1.45	1.04, 2.02	1.35	0.95, 1.90	1.32	0.97, 1.67

Abbreviations: IQ, the full-scale score; OR, odds ratio; CI, confidence interval.

^a^Model 1: Crude odds ratios;

^b^Model 2: Race, sex, socioeconomic status, highest maternal education, maternal age, gestational age, marital status, maternal smoking, hypertensive disorders during pregnancy, birthweight, feeding methods, maternal pregnancy BMI, BMI gain during pregnancy, parity and 5 minutes Apgar score were adjusted;

^c^Model 3: Generalized Estimating Equation model basing the same factors as model 2.

## Comments

To our best knowledge, this is the first study reporting that, in term newborn babies, exposure to high concentrations of bilirubin is inversely associated with obesity in children at age 7 years, without increasing the risk of low IQ.

It was well-known that bilirubin has toxic effects on developing neuronal tissues, and high serum bilirubin concentrations are associated with neurological dysfunction in newborn babies^6,7^. However, recent investigations reported on mild hyperbilirubinaemia having positive health effects by exerting anti-oxidant, anti-inflammatory activities^8–10^, increasing insulin sensitivity^10,18^ and regulating lipid synthesis^18,28^. Obesity, increasing all over the world, is a low-grade inflammatory disease^11,12^. Population based studies have documented that serum bilirubin levels are inversely associated with abdominal obesity in adults and adolescent^22,27,29,30^. However, these cross-sectional studies regarding obesity did not find associations to neonatal bilirubin levels. By using the prospective data from the U.S. CPP, we could document that exposure to bilirubin in the neonatal period was inversely associated with obesity in later life. The previous studies reported that the elevated bilirubin could increase insulin sensitivity^10,18^, reduce lipid synthesis^18,28^, and regulate the serum level of leptin, PPAR α and adiponectin^18^, then reduce the body weight. Thus, we refer that these factors may involve in the potential mechanism of the inverse correlation between bilirubin concentrations and childhood obesity.

Our results showed that the two subtypes of bilirubin have opposite associations to childhood obesity in term babies. These findings are consistent with one previous study reporting on the inverse relation of direct bilirubin/CB, rather than indirect bilirubin/UCB, to metabolic syndrome^15^. Furthermore, regarding our results, it seems that exposure to UCB, even on physiological levels (<13 mg/dl), is inversely associated with childhood obesity in our study subjects, while serum CB seems to have a positive dose-response relationships with childhood obesity. As the underlying causes of jaundice in neonates may also be non-physiological, we excluded infants presenting pathological factors, still confirming the negative correlations between serum bilirubin levels in newborns and body mass index at age 7 years.

For the anti-oxidative and anti-inflammatory properties, bilirubin has been suggested as possible target to intervene cardio-metabolic disorders in future^31,32^. Regarding the concentrations of bilirubin presented in previous population based studies they refer to the normal ranges. In an experimental study infusing bilirubin directly to mice, Vera *et al*. found that moderate hyperbilirubinemia (1 mg/dl) resulted in a decrease in vascular oxidative stress^33^. Another experimental study using a Gunn rat model, inducing severe hyperbilirubinemia, showed that bilirubin was protecting for cardiac reperfusion injury. The bilirubin concentrations in this model were much higher than those observed in population based studies^34^. However, there was no evidence on this topic from natural exposure to high bilirubin level. The physiological exposure to bilirubin in the neonate is much higher than in any other period of life in humans. Using the data set of the CPP study period (1959–1966), when phototherapy was not used routinely for the treatment of neonatal jaundice, made it particularly suitable to study the exposure-effect relationships of untreated neonatal serum bilirubin and childhood obesity. Thus, our findings emphasizes the existence of a negative dose-response correlation between physiological jaundice in term babies and later childhood obesity, what may generate new hypothesis for interventional studies in the future to intervene obesity or obesity related diseases.

Regarding the role of lifelong exposure to increased serum bilirubin in Gilbert syndrome patients^31,32,35^, one may speculate on protective effects of hyperbilirubinemia. However, in an experimental study in mice beneficial health effects of high bilirubin levels could not be determined^33^. In contrast our results, indicating the beneficial effect of bilirubin on childhood obesity in the neonatal period could be interpreted as a potentially epigenetic effect determined only in early life.

Our study has several limitations as it is a historic study performed 40 years ago. For the more active intervention to neonatal jaundice in current guideline, the relevance of our finding to the contemporary populations may be questioned. However, for the same reasons, the same study is unlikely to be duplicated nowadays. Thus, we believe our findings have useful implications in the future to intervene obesity.

In conclusion, in term newborn babies exposure to physiological level of bilirubin is inversely associated with childhood obesity in later life without increasing the risk of low IQ. Our findings may shed some light on investigations and interventions regarding the role of bilirubin in the prevention and treatment of obesity or obesity related diseases.

## Data Availability

The CPP data are publicly available through the U.S. National Archives (www.archives.gov/).
